# Pericardial effusion uncovering underlying hypothyroidism

**DOI:** 10.1002/ccr3.3857

**Published:** 2021-01-25

**Authors:** Anoosha Ponnapalli, Smit S. Deliwala, Yazan Zayed, Ghassan Bachuwa

**Affiliations:** ^1^ Department of Internal Medicine Michigan State University at Hurley Medical Center Flint MI USA

**Keywords:** hypothyroidism, myxedema, pericardial disease, pericardial effusion

## Abstract

Pericardial effusion may be the tipping point to unravel hypothyroidism. Large effusions may not correlate with severity of cardiovascular compromise. Medical therapy over surgical intervention is appropriate if no evidence of cardiac tamponade.

## Graphical abstract

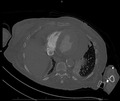

## 1 CASE HISTORY

Primary hypothyroidism has been associated with many undue adverse effects. Its cardiovascular manifestations include reductions in heart rate and contractility with the development of hypertension, angina, heart failure, and pericardial effusion consistent with hypothyroid heart. Early recognition and treatment can help mitigate these advanced features.[1]

**Question:** What are the cardiac implications of untreated hypothyroidism?

**Answer:** We present a case of pericardial effusion from primary hypothyroidism.

**FIGURE 1:**
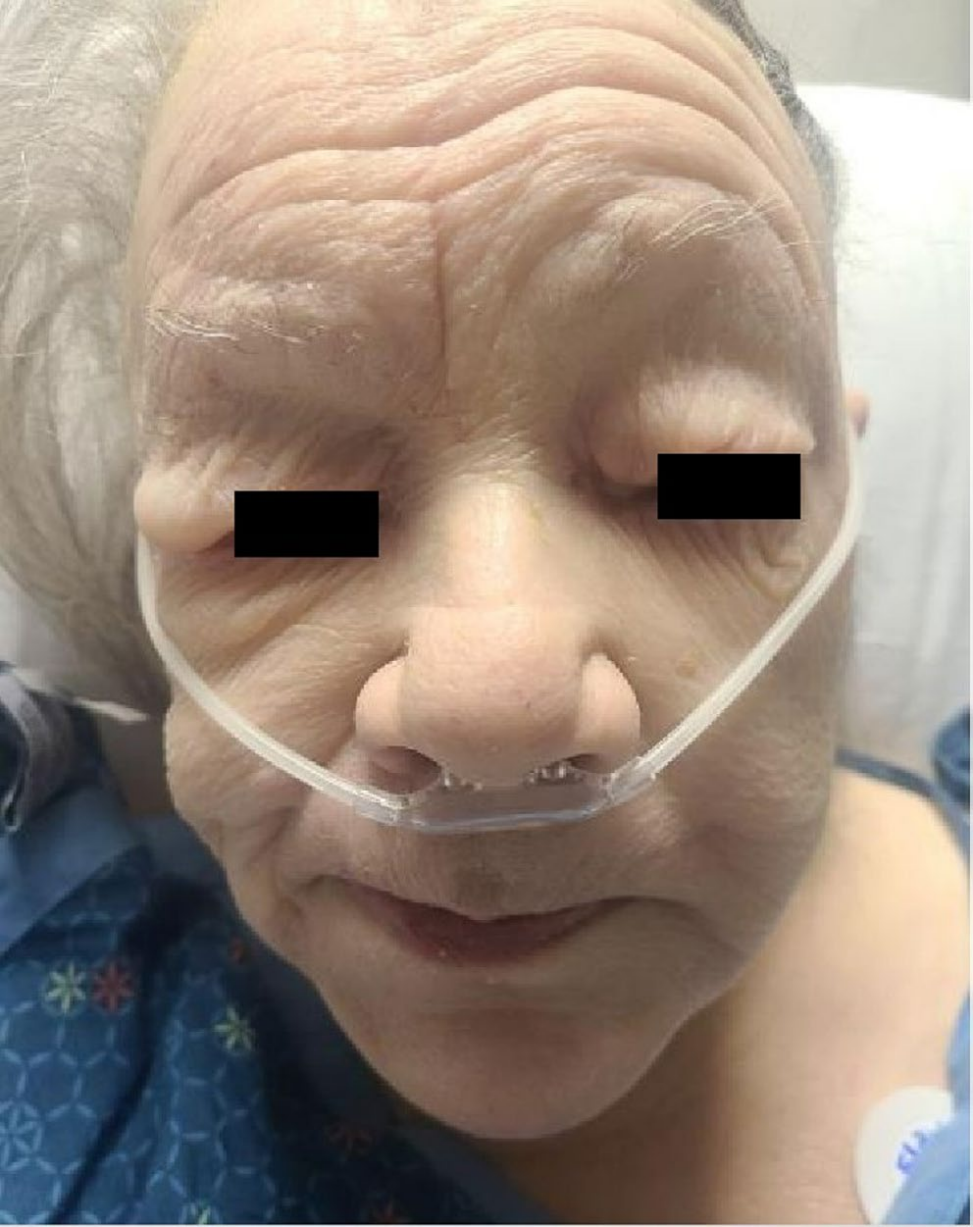
Myxedema demonstrating features of hypothyroidism

**FIGURE 2:**
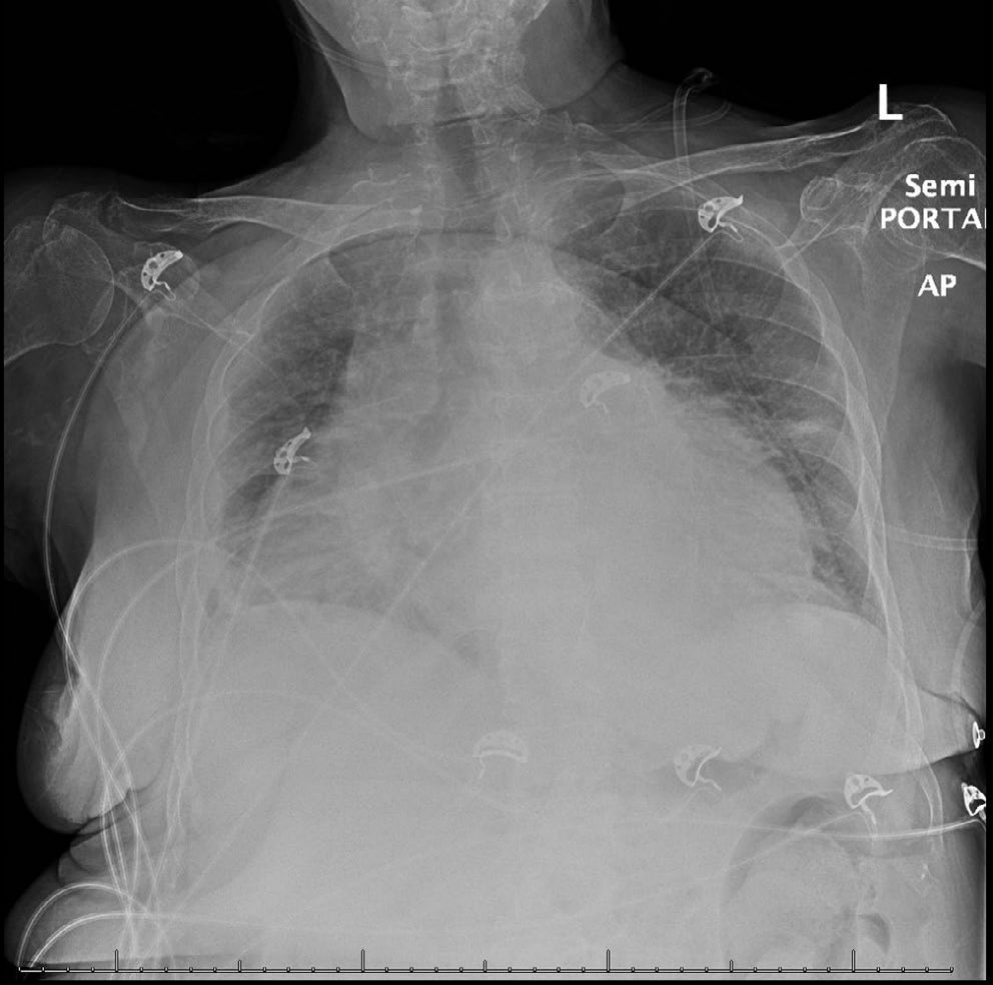
Chest radiograph demonstrating enlarged cardiac silhouette

**FIGURE 3:**
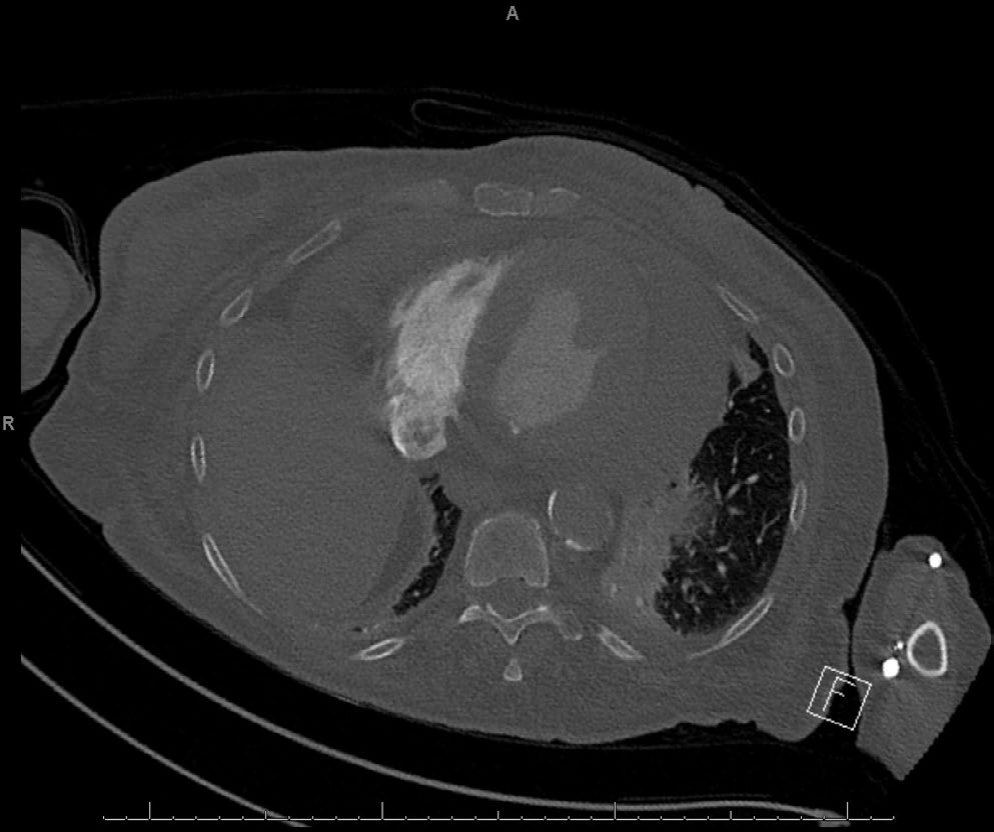
Computerized Tomography (CT) demonstrating large pericardial effusion

**FIGURE 4:**
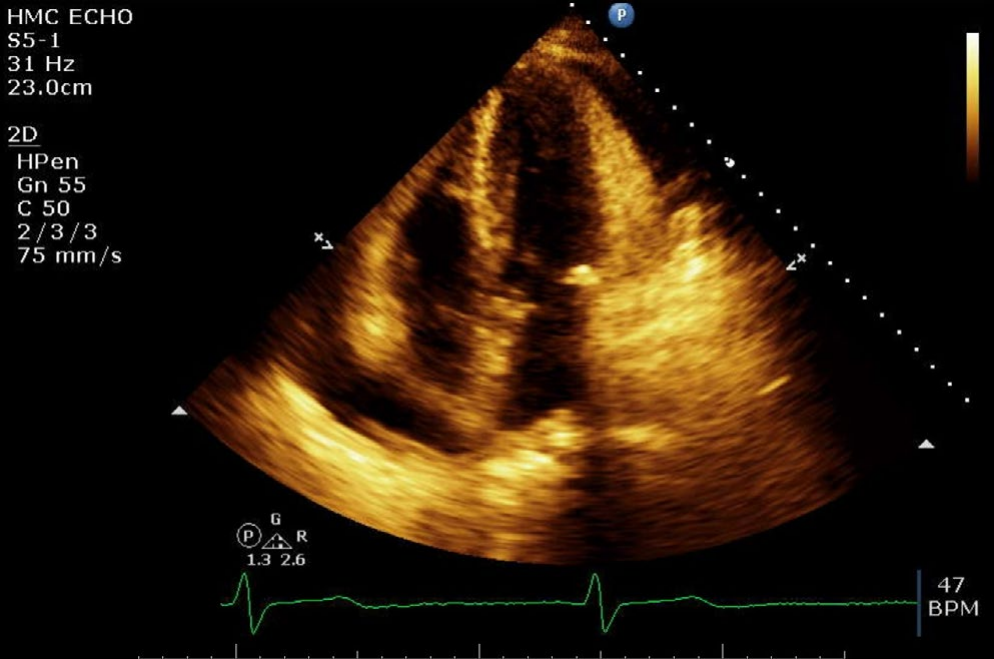
Transthoracic echocardiogram with four‐chamber view showing pericardial effusion

A 70‐year‐old woman with a history of chronic obstructive pulmonary disease (COPD), chronic arthritis with no documented hypothyroidism presented to the emergency department with dyspnea evolving over the last month, generalized weakness, and arthritic pain. Initial vitals were notable for bradycardia and hypoxia, with an oxygen saturation of 80% necessitating supplemental oxygen. She was awake, was appeared frail, and was not in acute distress. The examination revealed muffled heart sounds, jugular venous distention, and features consistent generalized myxedema (Figure 1). Initial workup revealed pancytopenia with macrocytosis and revealed an unremarkable chemistry panel with a brain natriuretic peptide (BNP) of 133.4 PG/ML. Imaging revealed cardiomegaly and pulmonary congestion on chest radiography (Figure 2), while computerized tomography (CT) chest with contrast revealed a moderate to large pericardial effusion (Figure 3). Thyroid‐stimulating hormone (TSH) was 102.94 UIU/ML (normal range 0.30‐5.50 UIU/ML) with a free T_4_ less than 0.1 NG/DL (normal range 0.9‐1.8 NG/DL). A transthoracic echocardiogram confirmed a large circumferential pericardial effusion without tamponade physiology and severe concentric left ventricular hypertrophy (Figure 4). She was initially treated with a single dose of intravenous levothyroxine 300 mcg, followed by 100 mcg daily. Also received hydrocortisone 50 mg every 8 hours. Following clinical improvement was transitioned to oral levothyroxine 100 mcg daily, and hydrocortisone was slowly discontinued. Repeat echocardiogram prior to discharge revealed significant improvement, showing only moderate effusion adjacent to the left ventricle. She was discharged on an oral course of levothyroxine with close follow‐up with a cardiologist.

## CONFLICT OF INTEREST

None to declare.

## AUTHOR CONTRIBUTIONS

Anoosha Ponnapalli: designed the manuscript, collected the data, and reviewed the manuscript. Smit S. Deliwala: collected the data, prepared the manuscript, and reviewed the manuscript. Yazan Zayed: reviewed the manuscript. Ghassan Bachuwa: reviewed the manuscript.

## ETHICAL STATEMENT

Informed consent was obtained from the patient for the publication of this case report.

## Data Availability

Data sharing was not applicable as no datasets were generated or analyzed for this article.
